# Quatrefoil light traps for free-swimming stages of cymothoid parasitic isopods and seasonal variation in their species compositions in the Seto Inland Sea, Japan

**DOI:** 10.1016/j.ijppaw.2022.12.002

**Published:** 2022-12-16

**Authors:** Hiroki Fujita, Kentaro Kawai, Diego Deville, Tetsuya Umino

**Affiliations:** Graduate School of Integrated Sciences for Life, Hiroshima University, 1-4-4 Kagamiyama, Higashi-Hiroshima, Hiroshima, 739-8528, Japan

**Keywords:** 16S rRNA, COI, DNA barcoding, Fish parasite, Life cycle, Manca, Optional intermediate host, Reproductive season

## Abstract

Cymothoid parasitic isopods infest a wide range of fish of different taxa living in marine, brackish, and freshwater environments. Most research on the reproductive season of Cymothoidae has been done by collecting or monitoring host fish afflicted with cymothoid parasites. However, collecting ecological data on cymothoid species that infest non-commercial or endangered fishes is complex and challenging. We used a quatrefoil light trap to investigate the seasonal change in species composition of cymothoid free-swimming stages in the Seto Inland Sea, Japan. We also collected preliminary data for efficient light-trap sampling and showed its effectiveness in cymothoid-related research. From October 2020 to December 2021, 613 cymothoid free-swimming stages were sampled monthly. All obtained individuals were identified as *Mothocya parvostis* (596), *Ceratothoa verrucosa* (12), and *Ceratothoa carinata* (5) by DNA barcoding using cytochrome *c* oxidase subunit I and 16S rRNA gene sequences. Based on the number of *M. parvostis* mancae collected each month, *M. parvostis* was anticipated to reproduce from June to December, with two reproduction peaks each year, and *C. verrucosa* and *C. carinata* were expected to reproduce in June, July, and September, and September and October, respectively. In addition, free-swimming juveniles were captured, presumably after they had left their optional intermediate hosts. Furthermore, the most effective time to harvest cymothoids with light traps may be during high tide on the night of the new moon. This study serves as a methodological framework for future research on cymothoids using light traps.

## Highlights

• Light trapping is useful method for studying the life cycle of cymothoid parasites.
• Light trapping is most efficient when conducted at the high tide of the new moon.
• The reproductive seasons of three cymothoids were estimated.
• *Mothocya parvostis* has optional intermediate hosts.

## Graphical abstract

**Figure:**
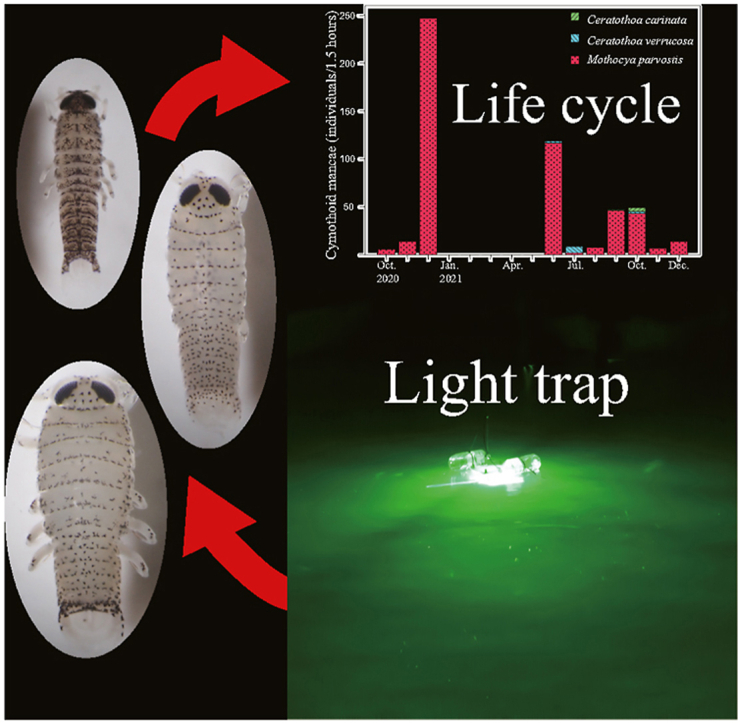

## 1 Introduction

Cymothoidae Leach, 1818 is a cosmopolitan parasitic isopod family that includes 363 species and 42 genera (Boyko et al., 2008). The hosts of these isopods include a wide range of fish taxa found in marine, brackish, and freshwater settings (Yamauchi, 2016). These parasites adhere to their hosts at four different locations: the opercular cavity, the buccal cavity, the abdominal cavity, and the body surface (Smit et al., 2014). During their life cycle, free-swimming mancae (larvae) infest the host fish and develop into adult cymothoids via juveniles, after which their sex switches from male to female (Smit et al., 2014). Cymothoid parasites are known to negatively impact fish in response to environmental changes (Östlund et al., 2005; Kawanishi et al., 2016), therefore, understanding the diversity, life cycle, and host-parasite relationships of these parasites are essential for understanding and conserving the ecosystem. Reproduction biology is important for understanding the life cycle of cymothoids, especially the dynamics of the free-swimming stages.

Due to the limited collecting methods for cymothoids, very few cymothoid species have been investigated for their reproduction. Most investigations focus on adult or juvenile parasites on collected host fishes (Bello et al., 1997). However, this procedure can only be applied to easily collectable host fishes. Alternative approaches for examining the reproduction of cymothoid species could be derived from other fish research using their planktonic eggs in the sea. For example, egg density of the black sea bream *Acanthopagrus schlegelii* (Bleeker, 1854) in the ocean is used to estimate their spawning season (Kawai et al., 2017, 2020), spawning grounds (Kawai et al., 2021), and spawning time (Kawai et al., 2022). Since cymothoids breed by releasing free-swimming mancae, it may be possible to determine the reproduction season by collecting mancae from the ocean. The free-swimming cymothoid stages can be caught using light traps (Jones et al., 2008; Saito et al., 2014).

In addition, species identification procedures for cymothoids can interfere with reproduction research. As cymothoids can only be identified based on morphological characteristics of adult females, their mancae, juveniles, and males need to be identified by molecular analysis (Fujita et al., 2021; Saito and Fujita, 2022; Saito et al., 2022). The reproductive season can be approximated by employing DNA barcoding to identify free-swimming stages captured by light traps and observing seasonal variation in appearance.

In this study, we investigated seasonal variation in species composition, abundance, and life stages of free-swimming cymothoids collected in the Seto Inland Sea where seven cymothoid species are distributed (Yamauchi et al., 2004; Yamauchi and Nagasawa, 2012; Nagasawa et al., 2014; Nagasawa and Tensha, 2016), and discussed the reproduction season and life cycle of each species. By integrating the light trap collecting method and DNA barcoding identification, this study serves as a methodological framework for investigating the reproduction and life cycles of cymothoids in their free-swimming stages.

## 2 Materials and methods

### 2.1 Sampling

**Fig. 1:**
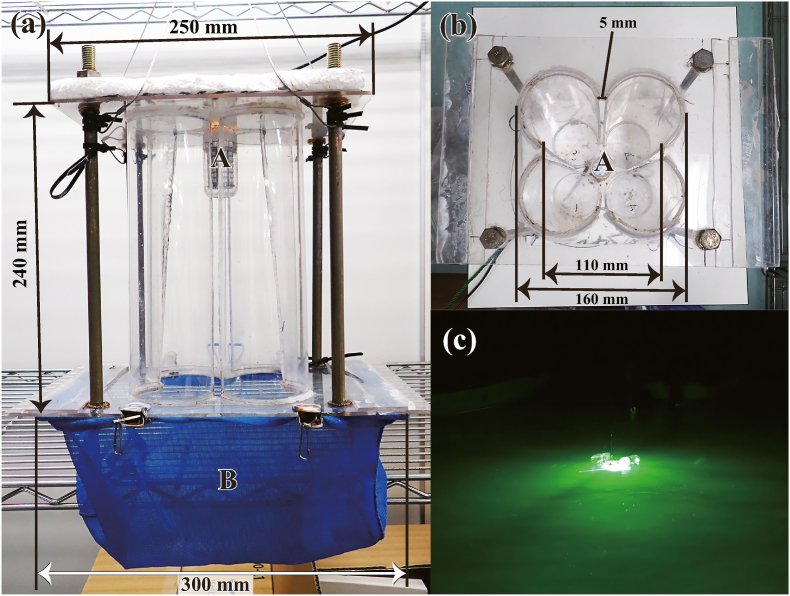
The quatrefoil light trap using in this study. (a): front view, (b): bottom view without net, (c): Light traps in use underwater. A: 15 W LED fishing light, B: Net to collect organisms in trap (0.5 mm mesh).

**Fig. 2:**
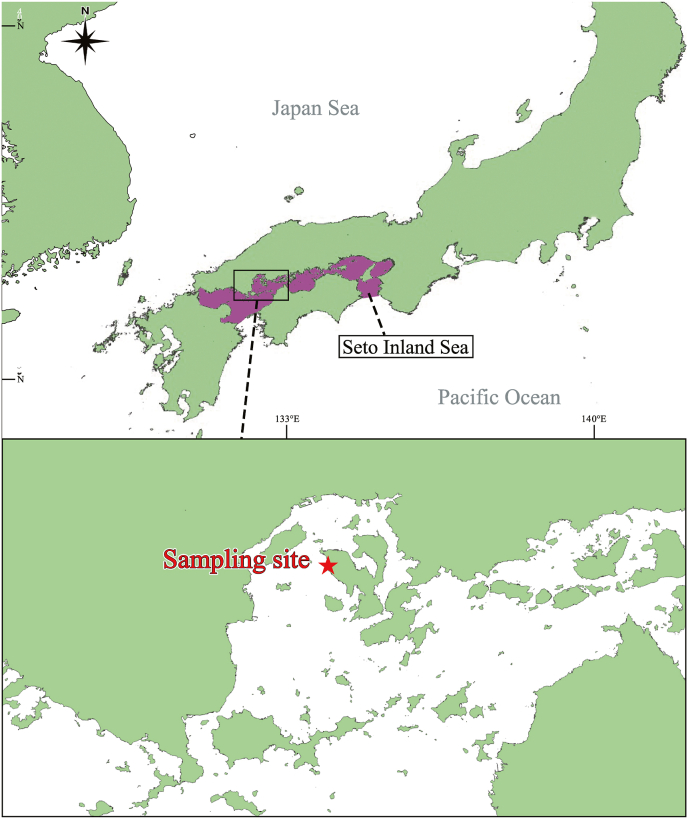
Map showing location of the Seto Inland Sea and sampling site, where light trap sampling was performed.

A quatrefoil light trap (height: 204 mm, width: 250 mm, net mesh size: 0.5 mm; Fig. 1), fabricated following Floyd et al. (1984), was used for collecting free-swimming cymothoids. Sampling was performed from October 2020 to December 2021 on the west shore of Nomijima Island, Etajima City, Hiroshima Prefecture, Japan (34°13′49.8"N 132°23′17.2"E; Fig. 2) at night. We count 30 min of lighting as one sampling operation.

The number of cymothoids collected on December 9th, 12th, and 15th of 2020 was compared at different tide levels to assess the effect of tidal range on cymothoid numbers and the best tidal level for their sampling. Each low tide, 1/3 tide, 2/3 tide, and high tide had one sampling operation.

We analyzed the number of cymothoids collected particularly from November 15th to December 15th in 2020 within the sampling period to assess the variation of collected cymothoids and the best days for sampling over the course of a month. Three sampling operations were carried out during the 90 min preceding and following the high tide every 3 days.

Based on the results of the above two surveys, monthly sampling was carried out to determine the seasonal fluctuation in the species composition of free-swimming cymothoids. Each month, three sampling operations were carried out during the 90 min preceding and following the high tide of the new moon.

After each operation, the trap was pulled up after turning the lights off. Plankton trapped in the net at the bottom of the trap was then packed in bottles and transported on ice to our laboratory. Following Bruce (1985), cymothoids were separated from the collected plankton. Each specimen was measured for its total length and then fixed in 99.5% ethanol. The life stages of cymothoids were identified following Aneesh et al. (2016); in particular, mancae were distinguished from juveniles by the absence of pereopods 7 (presence in juveniles). Water temperature was measured using a HOBO pendant logger UA-002-64 (Onset, MA, USA) placed at the sampling sites at a depth of 1 m. All applicable international, national, and institutional guidelines for the care and use of animals were strictly followed. All animal sample collection protocols complied with the current laws of Japan.

### 2.2 Molecular identification

Total DNA was extracted from the pereopods of the cymothoid specimen using the alkaline lysis method according to the recommended protocol for KOD FX Neo DNA polymerase (Toyobo, Osaka, Japan). The pereopod was mixed with 18 μL of NaOH (50 mM) and incubated at 95 °C for 10 min. The tubes containing 2 μL of Tris-HCl (1 M, pH 8.0) were extensively vortexed and centrifuged at 12,000 rpm for 5 min. The supernatant was separated and frozen at −30 °C until use in the polymerase chain reaction (PCR). The primers LCO1490-Fujita (5′-ACAAAHCATAAAGATATT-3′) and HCO2198-Fujita (5′-ACTTCDGGGTGRCCRAAAAATC-3′) manually modified based on Folmer et al. (1994) were used to amplify partial cytochrome *c* oxidase subunit I (COI) sequences. Partial 16S rRNA sequences were amplified using primers 16Sar (5′-CGCCTGTTTAACAAAAACAT-3′), and 16Sbr (5′-CCGGTCTGAACTCAGATCATGT-3′) (Simon et al., 1994). The total volume for each PCR cocktail was 8.1 μL, which included 1 μL of DNA, 0.78 μL of ultrapure water, 4.06 μL of 2 × PCR buffer, 1.62 μL of dNTP mix, 0.24 μL of each primer (10 μM solutions), and 0.16 μL of KOD FX Neo DNA polymerase. The thermocycler profile for COI consisted of an initial denaturation at 94 °C for 2 min; followed by 35 cycles of denaturation at 98 °C for 10 s, annealing at 50 °C for 30 s, and extension at 68 °C for 45 s; and a final extension at 68 °C for 7 min. The thermocycler profile for 16S rRNA consisted of initial denaturation at 94 °C for 2 min; followed by 35 cycles of denaturation at 98 °C for 10 s, annealing at 50.5 °C for 30 s, and extension at 68 °C for 30 s; and a final extension at 68 °C for 7 min. Dye-terminator methods were used to sequence PCR products with an ABI 3130xl Genetic Analyzer (Applied Biosystems, CA, USA). The determined sequences were deposited in GenBank (LC741450–LC741549, LC741603–LC742126). Basic local alignment search tool (BLAST) was run on each sequence in the National Center for Biotechnology Information database. We established confidence values for identification with BLAST (≥99% similarity and an E-value = 0.0).

Prior to the analysis of the free-swimming cymothoids, the COI and 16S rRNA sequences of an adult male-female pair of *Ceratothoa carinata* (Bianconi, 1869) collected from the buccal cavity of a Japanese scad *Decapterus maruadsi* (Temminck and Schlegel,1844) caught in Sagami Bay, Kanagawa, Japan on April 4, 2022, were deposited in GenBank as reference sequences (COI: LC724050, LC724049; 16S rRNA: LC724051, LC724052). This cymothoids were identified as *C. carinata* because of the features: the contiguous and swollen antennular bases, the dorsal pereon surface with medial longitudinal ridge present, and the concave posterior margin on the pleotelson (Hadfield et al., 2016).

## 3 Results

**Fig. 3:**
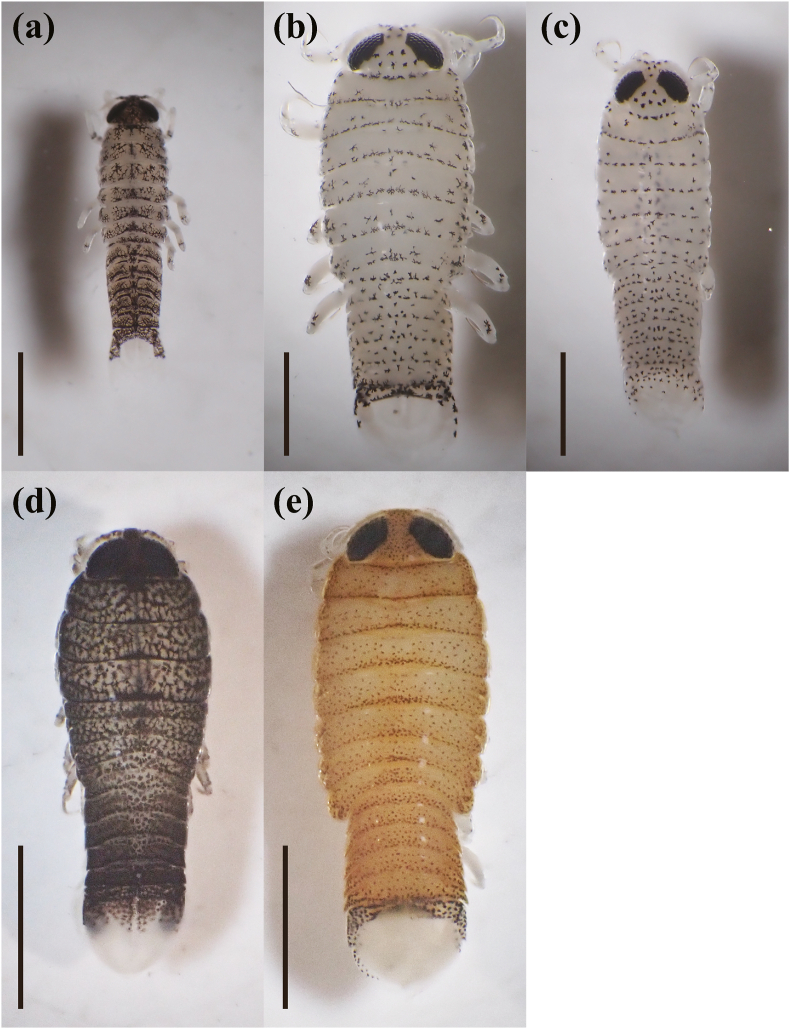
Dorsal views of cymothoid free-swimming stages collected by the light trap. (a) and (d): *Mothocya parvostis*, (b) and (e): *Ceratothoa verrucosa,* (c): *Ceratothoa carinata.* (a)–(c): mancae, (d) and (e): juveniles. Scale bars indicate (a)–(c): 1 mm, (d) and (e): 3 mm.

**Fig. 4:**
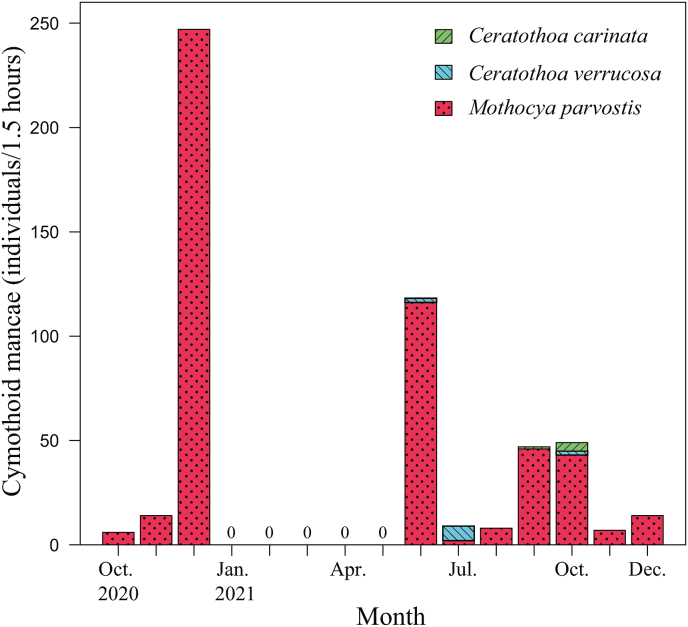
Number of cymothoid mancae collected in each month from October 2020 to December 2021. Dot bars (red) indicate *Mothocya parvostis*, diagonal right pattern bars (blue) indicate *Ceratothoa verrucosa*, diagonal left pattern bars (green) indicate *Ceratothoa carinata*. (For interpretation of the references to colour in this figure legend, the reader is referred to the Web version of this article.)

**Fig. 5:**
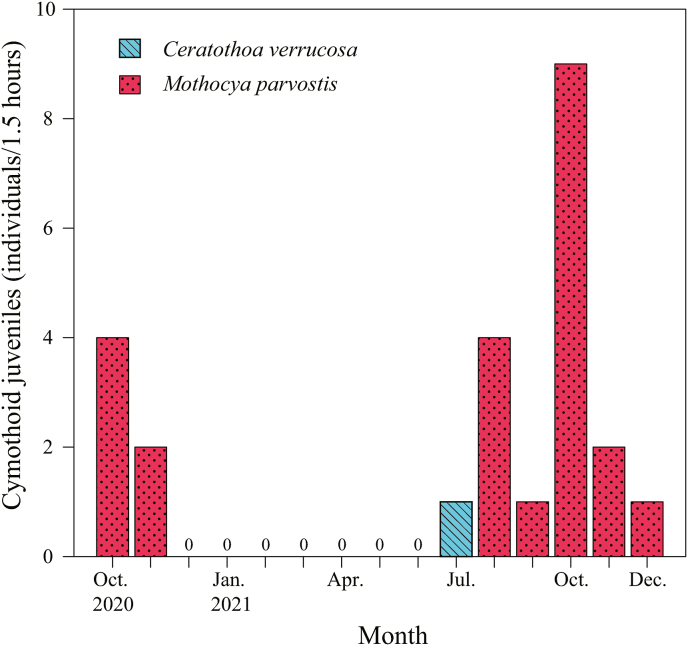
Number of cymothoid juveniles collected in each month from October 2020 to December 2021. Dot bars (red) indicate *Mothocya parvostis* and diagonal right pattern bars (blue) indicate *Ceratothoa verrucosa*. (For interpretation of the references to colour in this figure legend, the reader is referred to the Web version of this article.)

**Fig. 6:**
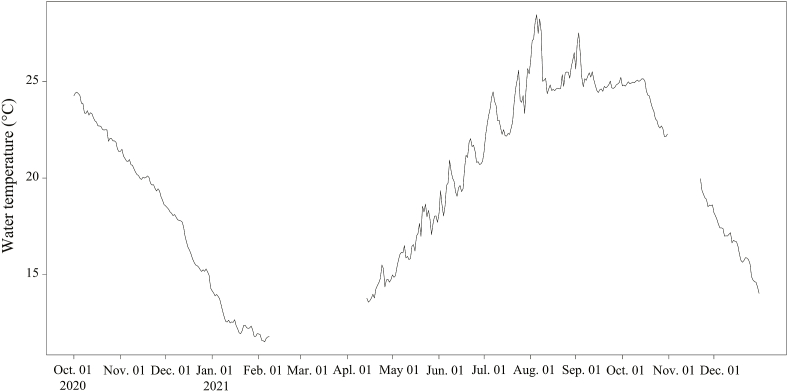
Temporal variation in water temperature from October 2020 to December 2021. The gap in data is due to faulty logging equipment.

We collected 589 mancae and 24 juveniles of Cymothoidae with the light trap. All individuals were successfully identified by DNA barcoding, with 596 individuals identified as *Mothocya parvostis* Bruce (1986), 12 individuals as *Ceratothoa verrucosa* (Schioedte and Meinert, 1883), and five individuals as *C. carinata* (Table 1; Fig. 3; Supplementary Table S1). The mancae of *M. parvostis* were collected from June to December with abundance peaking in June and from September to December (Fig. 4). The juveniles of *M. parvostis* were collected from August to December, with the highest abundance in October (Fig. 5). The mean total length of *M. parvostis* did not overlap between mancae and juveniles (Table 1). The mancae of *C. verrucosa* were collected in June, July, September, and October, with the highest abundance in July (Fig. 4). A single *C. verrucosa* juvenile was collected in July 2021 (Fig. 5). The total length of *C. verrucosa* juveniles was much larger than that of mancae (Table 1). No juvenile *C. carinata* were collected, and mancae were collected in September and October 2021 (Fig. 4, Fig. 5). No cymothoids were collected from January to May. The dominant species for all months, except July 2021, was *M. parvostis*. In July 2021, *C. verrucosa* was the dominant species. The water temperatures at the sampling site ranged from a minimum of 11.24 °C to a maximum of 33.43 °C (Fig. 6). There are no data for water temperature from February 9th to April 13th and from October 31st to November 21st^,^ 2021 due to challenges with the logger.

**Table 1:** Results of sampling of cymothoid free-swimming stages collected by the quatrefoil light trap, and molecular identification using cytochrome *c* oxidase subunit I (COI) and 16S rRNA genes. Data for each individual are presented in Supplementary Table S1.

	Mancae		Juveniles		Accession No.		Reference accession No.	
Species	Number of samples	Total length (mm)	Number of samples	Total length (mm)	COI	16S rRNA	COI	16S rRNA
*Ceratothoa carinata*	5	4.25 ± 0.179 (3.98–4.5)	–		LC741960, LC741976, LC741977, LC741986, LC741987	LC742065, LC742069, LC742070, LC742084, LC742085	LC724049, LC724050	LC724051, LC724052
*Ceratothoa verrucosa*	11	3.95 ± 0.415 (3.24–4.72)	1	9.17	LC741856, LC741916–LC741921, LC741985	LC742051, LC742056, LC742057, LC742068	LC159556, LC160317	LC159444
*Mothocya parvostis*	573	3.06 ± 0.231 (2.31–3.96)	23	8.58 ± 0.947 (6.16–9.76)	LC741450–LC741549, LC741603–LC741855, LC741857–LC741915, LC741922–LC741959, LC741961–LC741975, LC741978–LC741984, LC741988–LC742004	LC742005–LC742050, LC742052–LC742055, LC742058–LC742064, LC742066, LC742067, LC742071–LC742083, LC742086–LC742126	LC159573, LC412904, LC549123, LC549124, LC549127, LC549140, LC549142, LC549143, LC549145, LC549148, LC549149, LC549150	LC159462, LC416622, LC159462, LC549152, LC549172, LC549177

**Fig. 7:**
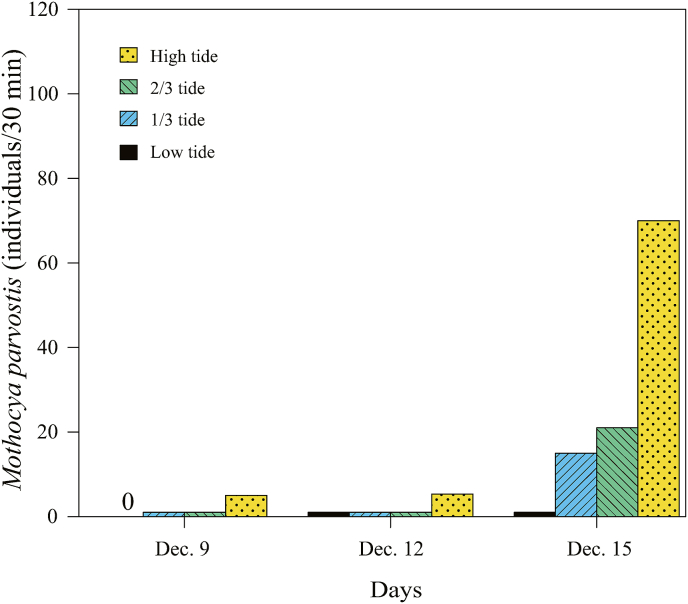
Number of *Mothocya parvostis* collected at tidal levels: low tide, 1/3 tide, 2/3 tide, and high tide during the three days of sampling.

**Fig. 8:**
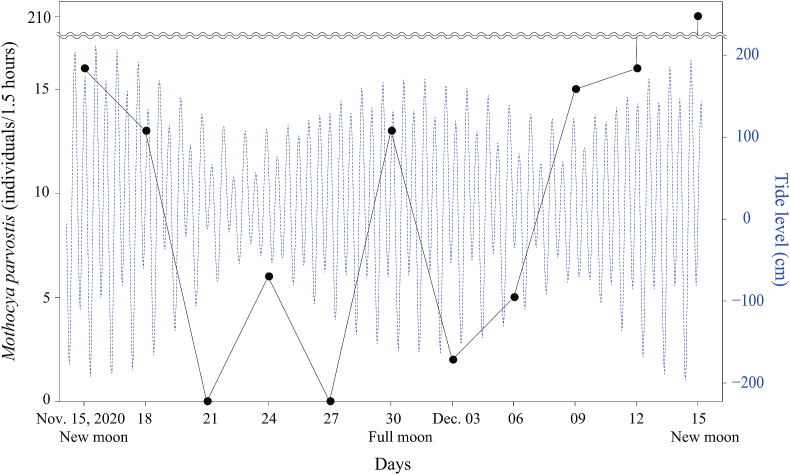
Number of *Mothocya parvostis* collected on each sampling date (solid line) and tidal levels (broken line) from November 15 (new moon) to December 15 (new moon).

DNA barcoding identified all cymothoids collected from November to December 2020 as *M. parvostis*. When the number of *M. parvostis* collected was compared to the daily tide level (low tide, 1/3 tide, 2/3 tide, and high tide), the species appeared to be most abundant on all survey days at high tide (Fig. 7). The number of *M. parvostis* collected on each sampling day increases during spring tide and decreases during neap tide (Fig. 8). Especially abundant *M. parvostis* were obtained during a spring tide at the new moon.

## 4 Discussion

In this study, we successfully performed quantitative collections of three free-swimming cymothoid species using a quatrefoil light trap. Although the quatrefoil light trap was developed for collecting fish larvae (Floyd et al., 1984), the method, together with DNA barcoding identification, is useful for studying cymothoid life cycles. Incidentally, free-swimming mancae die within 1 week–10 days after detaching from the brood pouch of cymothoid females if they could not find any hosts (Menzies et al., 1955; Hatai and Yasumoto, 1980; Sandifer and Kerby, 1983). Their starvation resistances affect estimating accuracy of the reproduction month of cymothoids using this method.

Hickford and Schiel (1999) reported that the number of fish larvae collected by the light trap method negatively correlated with lunar illumination, with fish larvae being more abundant at new moons than at full moons. In this study, we documented changes in the number of *M. parvostis* collected by light trapping over a month. Free-swimming *M. parvostis* appeared more abundant at spring tides, especially at new moons than at full moons. The number of free-swimming cymothoid individuals collected by light trapping may be affected by both lunar irradiance and tidal effect. Since mancae of Cymothoidae use surface tension to float on the water's surface (Adlard and Lester, 1995), a higher number of mancae floating offshore may reach the traps during spring tides when the difference in tidal range is greater. In addition, the number of *M. parvostis* collected by with a light trap was lowest at low tide and highest at high tide. This method of collecting cymothoids with a light trap was considered most efficient at high tide on a new moon night. However, because the collected number of zooplankton depends on various physical and chemical factors such as flow speed, turbidity and depth (Hickford and Schiel, 1999; Mcleod and Costello, 2017), the factors affecting the number of cymothoids collected by the light trap method need to be examined in more detail.

In DNA barcoding using a database such as GenBank, sequences labeled with incorrect species names are problematic for species identification (Shen et al., 2013). However, the reference sequences (Table 1) used in this study can be trusted for the following reasons. The COI (LC159573, LC412904, LC549122–LC549151) and 16S rRNA sequences (LC159462, LC416622, LC549152–LC549178) of *M. parvostis*, were morphologically and genetically proven to be derived from *M. parvostis* by Fujita et al. (2020, 2022). The COI (LC159556, LC160317) and 16S rRNA sequences (LC159444) of *C. verrucosa*, were registered by Hata et al. (2017). They did not report the morphology of these individuals. However, these two individuals were collected from the buccal cavities of the Crimson seabream *Evynnis tumifrons* (Temminck and Schlegel, 1843) and the red seabream *Pagrus major* (Temminck and Schlegel, 1844); *C. verrucosa* is the only cymothoid species reported in the buccal cavities of these fish species (Yamauchi, 2016). The COI (LC724050, LC724049) and 16S rRNA sequences (LC724051, LC724052) of *C. carinata*, were sequenced from the female and male morphologically identified in this study.

Seven species of cymothoids are reported in the Seto Inland Sea: *Anilocra clupei* Williams and Bunkley-Williams, 1986, *C. carinata*, *C. verrucosa*, and *Elthusa sacciger* (Richardson, 1909), *M. parvostis* and *Nerocila japonica* Schioedte and Meinert, 1881, and *N. phaiopleura* Bleeker, 1857 (Yamauchi et al., 2004; Yamauchi and Nagasawa, 2012; Nagasawa et al., 2014; Nagasawa and Tensha, 2016). In this study, three species of Cymothoidae, namely *M. parvostis*, *C. verrucosa*, and *C. carinata*, were collected with a light trap from June to December and identified by DNA barcoding. In the collected cymothoids, both manca and juvenile *M. parvostis* were substantially more abundant. In addition, no cymothoids were collected from January to May. It is possible that the lower water temperatures (lower than 15 °C; Fig. 6) during this period either prevented cymothoid reproduction, or reduced the activity of mancae, thus reducing the area over which they could spread.

Inoue (1941) proposed the life cycle of *M. parvostis* (previously identified as *Irona melanosticta* Schioedte and Meinert, 1884; Bruce, 1986) and suggested that *M. parvostis* mancae are released from adult females of *M. parvostis* around June. In addition, *M. parvostis* mancae infestation on juveniles of *A. schlegelii* was observed from June to August (Fujita et al., 2020), on juveniles of the cobaltcap silverside *Hypoatherina tsurugae* (Jordan and Starks, 1901) from July to October, and on juveniles of the yellowfin seabream *Acanthopagrus latus* (Houttuyn, 1782) from October to December (Fujita et al., 2022). In this study, free-swimming mancae of *M. parvostis* were collected from June to December, and not from January to May. This result suggests that *M. parvostis* reproduce in the Seto Inland Sea from June to December. However, the number of collected mancae varied, with higher numbers collected in December 2020, June 2021, September 2021, and October 2021. In addition, only a few *M. parvostis* mancae were collected in July, the only month that the species was not dominant. Therefore, *M. parvostis* likely has two reproduction peaks per year, one in spring and another in autumn.

Sanada (1938) collected a total of 120 *C. verrucosa* individuals from *P. major* over a 10-month period in the Seto Inland Sea, and after observing mancae in the brood pouches of several females at a specific time of year he concluded that *C. verrucosa* mancae leave the brood pouch of females in late August. However, this does not mean that females do not release mancae in other seasons. In this study, *C. verrucosa* mancae were collected in June, July, and October, suggesting that *C. verrucosa* reproduction occurs over a wider period than the one proposed by Sanada (1938).

Despite numerous taxonomic studies, the life cycle and reproduction of *C. carinata* remain unknown. Our specimens collected in September and October 2021 are the first records of free-swimming *C. carinata* mancae. The collection suggests that *C. carinata* may reproduce in September and October. However, because *C. carinata* was not observed in October 2020, and only a small number of individuals was collected overall, further investigation is required to clarify its reproduction season.

Some cymothoid species, such as *A. clupei*; *A. pomacentri* *Bruce, 1987*; *M. parvostis*; *Nerocila acuminata* Schioedte and Meinert, 1881; *Olencira praegustator* (Latrobe, 1802); and *Telotha henselii* (Martens, 1869), have optional intermediate hosts besides final hosts (Adlard and Lester, 1995; Lindsay and Moran, 1976; Segal, 1987; Taberner et al., 2003; Fogelman and Grutter, 2008; Fujita et al., 2020; Fujita, 2022). In the case of *M. parvostis*, Fujita et al. (2020, 2022) hypothesized that juveniles leave optional intermediate hosts (*A. schlegelii*, *H. tsurugae*, and *A. latus* juveniles) to find the Japanese halfbeak *Hyporhamphus sajori* (Temminck and Schlegel, 1846) as final host. However, the free-swimming period of cymothoids after leaving optional intermediate hosts has not been proved. In this study, 24 free-swimming juveniles of *M. parvostis* were collected over an extended period. In addition, the body length of collected *M. parvostis* ranged from 2.31 to 3.96 mm in mancae and from 6.16 to 9.76 mm in juveniles with no intermediate-sized individuals collected. Therefore, the observation of free-swimming cymothoid juveniles in this study may not be accidental and proves that cymothoids have free-swimming periods after detaching from optional intermediate hosts. In addition, one juvenile of *C. verrucosa* was collected, raising the possibility that *C. verrucosa* has an optional intermediate host. Future studies are needed to clarify what fish species are infested with these free-swimming cymothoid juveniles before detaching, for example, using diet analysis with DNA metabarcoding.

Each juvenile fish reported as optional intermediate hosts of *M. parvostis* appears in the surf zone and is infested by *M. parvostis* at a different season; *A. schlegelii* is infested from June to September (Kawai et al., 2019; Fujita et al., 2020), *H. tsurugae* is infested from July to October, and *A. latus* is infested from October to January (Fujita et al., 2022). Although mancae infest each fish just after metamorphoses from larvae to juveniles and then mancae become juveniles on the hosts (Fujita et al., 2020, 2022), the optional intermediate hosts of *M. parvostis* are present in the surf zone during the entire reproduction season of the species. This is considered advantageous in increasing the parasitic efficiency of *M. parvostis* on the final host, *H. sajori*; the prevalence of *M. parvostis* on *H. sajori* is approximately 50% (Kawanishi et al., 2016; Fujita et al., 2020), which is higher than that of other cymothoids (e.g., *C. verrucosa*: 18.6%, *N. phaiopleura*: approximately 10–30%) (Nagasawa and Tensha, 2016; Ohtani et al., 2021). This high prevalence may be maintained by using suitable optional intermediate hosts different for each season.

In this study, we verified the utility of a new research method for the life cycles of cymothoids combined with quantitative light trap collection and DNA barcoding identification. We observed and recorded the reproductive season of three cymothoid species and provide evidence supporting the hypothesis that *M. parvostis* juveniles have a free-swimming period after leaving optional intermediate hosts. Thus, combining quantitative collection of free-swimming cymothoids by light trapping and species identification by DNA barcoding is an effective method for clarifying reproduction, distribution, and morphology of the free-swimming stages of cymothoids.

## Author contributions

HF designed and executed the experiments, collected samples, and wrote the manuscript. KK and DD performed data analyses, provided useful ideas on research design, and edited the manuscript. TU acted as supervisor, provided useful ideas on research design, and edited the manuscript. All authors have reviewed and approved the final manuscript.

## Declaration of competing interest

HF, KK, DD, and TU declare that they have no conflict of interest.
